# Magnetic Resonance Spectroscopy of the Brain in Alcohol Abuse

**Published:** 1995

**Authors:** George Fein, Dieter J. Meyerhoff, Michael W. Weiner

**Affiliations:** George Fein, Ph.D., is a professor in the department of psychology at the School of Medicine, University of California, San Francisco, and a research career scientist in the Department of Veterans Affairs Hospital, San Francisco, California. Dieter J. Meyerhoff, Dr.rer.nat., is an assistant professor of radiology at the School of Medicine, University of California, San Francisco. Michael W. Weiner, M.D., is a professor of medicine and radiology at the School of Medicine, University of California, San Francisco, and director of the Magnetic Resonance Center in the Department of Veterans Affairs Hospital, San Francisco, California

**Keywords:** Magnetic resonance imaging, magnetic resonance spectroscopy, AOD abuse, brain, cell membrane, AOD tolerance, neuroimaging

## Abstract

Magnetic resonance (MR) technology produces data on brain structure and activity without relying on radiation or invasive surgery. Magnetic resonance imaging (MRI) creates images, and magnetic resonance spectroscopy (MRS) produces spectra based on the ability of atomic nuclei in tissues to absorb and release pulses of energy. MRS studies of alcohol in the brain reveal that only a portion of the alcohol in the brain can be detected by MR technology, suggesting that alcohol there exists in multiple pools. The pools not visible using MRS is hypothesized to be bound to cell membranes. Indirect evidence from MR studies of chronic alcohol abusers suggests that tolerance to alcohol’s effects results in an increased rigidity of cell membranes that forces more alcohol to remain in the MR-visible pool (i.e., the pool not bound to membranes) compared with alcohol in the brains of nontolerant people.

Alcohol produces many of its effects in humans by acting on the brain. The ways in which alcohol affects brain function, however, have yet to be linked with neurochemical and neurophysiological processes. And many research methods that could elucidate these mechanisms are too invasive to conduct in humans. Brain research has received a boost, however, from the increasing diversity and usefulness of imaging techniques, which provide methods of peering inside the living human brain, and even revealing interactions between brain molecules, without invasive surgery.

During the past 10 to 15 years, one such technique, magnetic resonance (MR) technology, has advanced dramatically, creating new avenues for producing images of (i.e., imaging) brain structure and function in living organisms (i.e., in vivo). Magnetic resonance imaging (MRI) has become the preferred method by which researchers generate images of the central nervous system (CNS) because of its noninvasive nature and its finely detailed images of CNS tissues. Both magnetic resonance spectroscopy (MRS) and magnetic resonance spectroscopic imaging (MRSI) (which combines imaging and spectroscopy) permit scientists to observe the chemical compound alcohol in the living brain. These technologies allow researchers to determine how alcohol is distributed throughout the brain and whether this distribution helps explain alcohol’s effects on brain function; how long (after consumption) it takes alcohol to be distributed to various brain regions (i.e., the time-course); and whether alcohol in the brains of people tolerant to alcohol’s effects is altered with regard to magnitude, anatomic distribution, or time-course.

This article briefly describes the history and science of MR technology and explains its use in imaging alcohol in the brain. The article also reviews results indicating that only a portion of the alcohol in the brain after consumption is visible using MRS or MRSI, explaining that this partial visibility may provide a clue as to how alcohol interacts with brain cells and how this interaction may differ in people with physiological tolerance to alcohol’s effects. After outlining a method for testing an explanation of alcohol’s partial visibility and reviewing the preliminary results, the article discusses the potential importance of these observations for advancing our understanding of the development and chronic nature of alcoholism in humans.

## Evolution of Magnetic Resonance Technology

**Figure 1:**
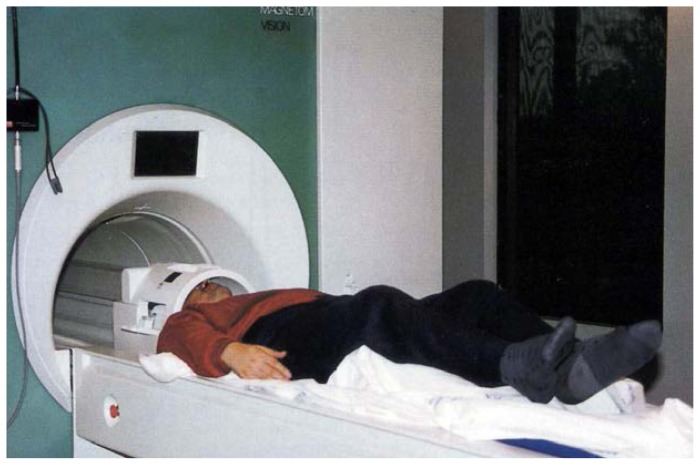
A subject is being prepared for a magnetic resonance examination. The subject lies in front of the magnetic resonance instrument on a movable bed. His head is encircled by an electronic device, which serves both to transmit low-energy radio frequency (RF) waves to hydrogen or phosphorus nuclei in the head and to receive the energy emitted from these nuclei. For the examination, the bed on which the subject lies is moved into the tunnel so that the magnet completely surrounds the subject’s head. The magnetic field affects all nuclei in the body so that they will absorb and emit RF energy.

The phenomenon of nuclear magnetic resonance was first discovered in 1946 independently by Felix Bloch at Stanford University and Edward Purcell at Harvard University (Bloch et al. 1946; Purcell et al. 1946). The discovery was of such farreaching importance that both researchers received the 1952 Nobel prize in physics for their work. Methods were first developed to exploit this phenomenon in physics; later, researchers in chemistry used MR technology to evaluate the structure and composition of organic and other molecules important to living organisms. In 1973, Lauterbur first demonstrated the ability of magnetic resonance to produce two-dimensional images of objects (Lauterbur 1973). Methodology advanced rapidly, with the eventual development in the early 1980’s of superconducting magnets that were large enough to accommodate a human body (figure 1). Since then, clinicians have used MRI routinely to obtain images of various organs in the human body. MRI produces images based on water’s distribution in the body. As radiologists have amassed information about variations in water’s properties in different classes of normal tissue (e.g., white versus gray matter in the brain)^1^ and in diseased tissue (e.g., tumors and multiple sclerosis plaques), they have designed MRI studies to accentuate these differences (e.g., to highlight the contrast between normal and specific types of diseased tissue).

Research into the use of MRS, which can evaluate molecules other than water, also started in the mid-1970’s, with the measurement of phosphorus spectra from isolated organs (see, for example, Garlick et al. 1977; Weiner et al. 1980) and intact animals (Ackermann et al. 1980). Scientists quickly realized that MRS can provide a window into metabolism and function in the human body without invasive procedures. Today, MRS is used primarily in biomedical research, but clinical applications are emerging, particularly in evaluating epilepsy, Alzheimer’s disease, metabolic muscle disorders, and brain tumors.

## The Workings of Magnetic Resonance Technology

Both MRI and MRS operate by affecting certain atoms (i.e., the fundamental units of matter) in the body. Atoms are made up of nuclei orbited by electrons. Different atoms (such as hydrogen and carbon) combine chemically to form compounds, which in turn compose the building blocks of body cells and tissues. Magnetic resonance detects the nuclei of certain atoms, such as hydrogen. MR instruments first use large magnets to prepare body tissues to be studied by aligning the protons of atoms in the tissues with the magnetic field. The machines then produce radio frequency (RF) waves that cause nuclei within these large magnetic fields to absorb and then emit energy, producing either images or spectra, depending on the specific technique (see also the article by Doria, pp. 261–265). In MRI, for example, the MR image of tissue shows the location in space (i.e., spatial distribution) of the hydrogen atom as it appears in water in various tissues. The hydrogen image varies in intensity based on how water’s concentration and properties change in different tissues.

**Figure 2:**
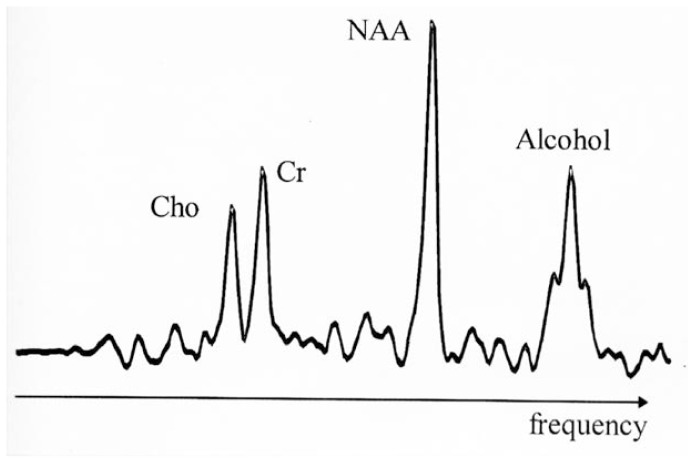
Magnetic resonance spectrum from hydrogen nuclei in a human brain (the signal from water, which would be much larger than the others, is suppressed). The peaks shown are from the three major signals visible by MRS^1^ in the normal brain, representing Cho,^2^ Cr,^3^ and NAA.^4^ The magnetic resonance spectrum was obtained after the subject had consumed alcohol; therefore, a fourth peak appears that originates from alcohol in the brain. This peak exhibits the typical triplet structure of alcohol and resonates at alcohol’s characteristic frequency at the right side of the spectrum. ^1^MRS = magnetic resonance spectroscopy; ^2^Cho = choline-containing compounds; ^3^Cr = creatinine-containing compounds; ^4^NAA = N-acetylaspartate.

Conversely, MRS produces its spectra from the energy emitted by hydrogen contained in compounds other than water. Hydrogen nuclei emit RF energy at different frequencies depending on the nuclei’s location in the body and the chemical compound in which they exist. In an MRS spectrum, signals of characteristic sizes and certain frequencies correspond to each of the hydrogen-containing compounds that are “visible” to the MR instrument. For example, several compounds that occur naturally in the brain appear as single peaks at specific points, or frequencies, along the spectrum. Alcohol appears as a triple peak at another frequency (figure 2). The area outlined by each peak is usually equal to the hydrogen concentration of its corresponding compound within the target tissue^2^ (although this is not the case with alcohol, as discussed below). The concentration tells researchers whether expected or unexpected levels of the detected compounds are present in the brain. MRS also can be directed to produce spectra from other atoms contained in living tissues (which do not contain hydrogen), such as phosphorus (discussed below).

### Resonance and Relaxation

**Figure 3:**
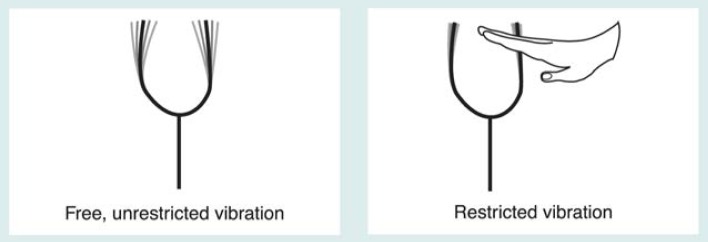
Tuning forks used to illustrate two basic phenomena of magnetic resonance. A tuning fork, when struck, resonates at a specific frequency in much the same way as an atom’s nucleus, when placed in a large magnetic field, absorbs and emits radio frequency (RF) energy at a specific frequency. The magnitude of the tuning fork’s vibration diminishes over time. Similarly, the amount of RF energy a nucleus emits after absorbing RF energy diminishes over time. The time required for the energy (i.e., the emission signal) to decrease by a certain percentage is called the T_2_ relaxation time. The T_2_ depends on the nucleus’s interaction with other nuclei in its surroundings (i.e., its molecular mobility). If a strong interaction exists that limits the nucleus’s mobility, the T_2_ relaxation time is short; if the mobility is not restricted, the T_2_ relaxation time is long. This outcome is analogous to a tuning fork sounding for a reduced period of time if its vibration is disturbed or restricted by touching it.

The effect of the tissues and compounds in the body (i.e., the nuclear environment) on magnetic resonance must be understood when interpreting findings. By altering the way the nuclei of atoms release energy, the nuclear environment affects the MRI or MRS outcome. To understand how MR technology elicits different degrees of response from atoms in different tissues, it helps to consider how a tuning fork works (figure 3). A tuning fork vibrates, or resonates, at a specific frequency, just as hydrogen nuclei in water or other compounds absorb RF energy at specific resonance frequencies. The vibration of a tuning fork slowly diminishes over time (i.e., it decays). Similarly, the magnitude of RF energy emitted by hydrogen nuclei decays over time. The duration (known in the field as T_2_)^3^ of this decay is called the relaxation time. If the ability of the tuning fork to vibrate is restricted (e.g., by someone touching it), the sound’s duration is reduced. Likewise, if the nuclei’s mobility is restricted, the relaxation time (i.e., the duration of decay) will be shortened.

### Magnetic Resonance Spectroscopic Imaging

**Figure 4:**
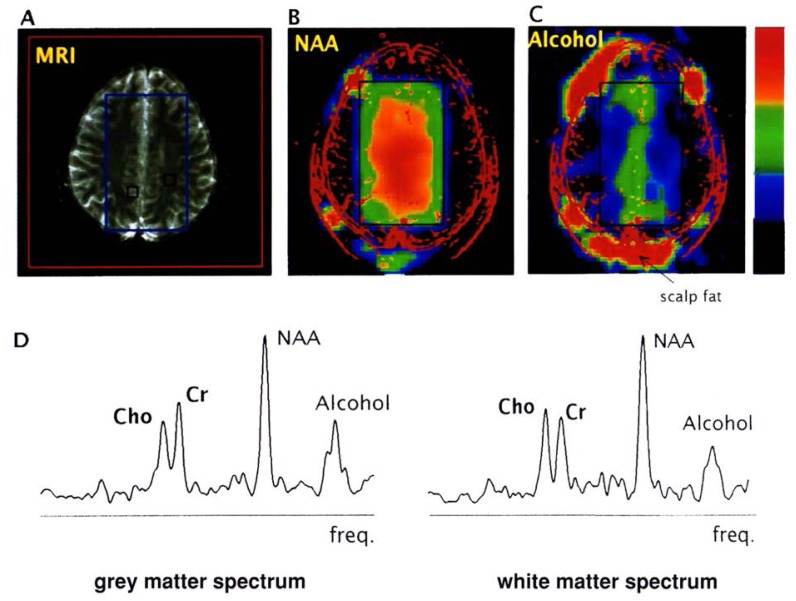
Results of a single hydrogen MRSI^1^ examination of a healthy subject who consumed alcohol before the study. (A) For comparison, a typical magnetic resonance image obtained from the hydrogen signal of water in the brain. Superimposed on this image is a rectangle corresponding to the region from which data are obtained during MRSI.^2^ The spectroscopy volume is selected well inside the brain, because an alcohol signal from brain close to the skin may be obscured by signals from scalp fat (seen around the perimeter of the image in C). (B) The spectroscopic image obtained from the normal distribution of the hydrogen signal of the chemical compound NAA^3^ located in the brain region outlined on image A. Within the spectroscopy rectangle (see color scale on right side), red indicates a high concentration of NAA; yellow and green represent successively lower concentrations. An outline of the head obtained from the image in A is superimposed in red lines to facilitate its comparison with the NAA image. (C) Spectroscopic image obtained from the hydrogen signal of alcohol in the rectangular region. Inside this spectroscopy volume, the highest alcohol concentration (shown by red and orange colors) is found in a specific tissue (i.e., gray matter) along the midline between the two brain hemispheres, whereas lower concentrations are found in another type of tissue (i.e., white matter).^*^ (D) Two representative spectra obtained from predominantly gray and white matter at locations indicated by black squares on the magnetic resonance image in A. Note the three characteristic brain resonances of hydrogen-containing compounds (labeled Cho,^4^ Cr,^5^ and NAA) and the typical peak from alcohol on the spectra’s right side. The stronger alcohol signal from the gray-matter spectrum, compared with that from the white-matter spectrum, suggests a higher concentration of alcohol in the gray matter. ^1^MRSI = magnetic resonance spectroscopic imaging; ^2^MRS = magnetic resonance spectroscopy; ^3^NAA = N-acetylaspartate; ^4^Cho = choline-containing compounds; ^5^Cr = creatinine-containing compounds. ^*^Gray matter is brain tissue composed mainly of the bodies of nerve cells and containing few nerve fibers. White matter is brain tissue composed mainly of nerve fibers and few nerve cell bodies.

A major drawback of early MRS studies was that only a single, small cube (i.e., volume) of tissue could be studied at one time, yielding a single spectrum. In recent years, ingenious methods have been developed for gathering spectra from multiple volumes of tissue simultaneously (for a review, see Meyerhoff 1994). This technology, MRSI, is based on the marriage of MRS to MRI methods and produces both structural images and spectra of corresponding volumes in the brain. In MRSI, researchers conceptually divide the tissue to be studied into a grid of volumes and gather a separate spectrum for each volume, or cube. The cube size used in an MRSI is dictated by the amount of tissue necessary to generate enough energy to produce a spectrum (approximately 1 cubic centimeter for hydrogen MRSI). The location of the cube of tissue represented by a particular MR spectrum can be shown on an accompanying MR image taken just before or after the MRS study (figure 4).

## The Brain Alcohol Signal Using MRS: Partial Visibility

The alcohol signal in MR spectra, such as those shown in figure 4, is used to calculate the alcohol concentration in a selected region of the brain. Research consistently shows that alcohol freely passes through the blood-brain barrier (see Chin 1979) and that alcohol levels in the blood and in the brain are roughly equal. Using MRS in subjects who had just consumed alcohol, investigators were surprised to find that the concentration reflected by the alcohol signal in the brain was much lower than the blood alcohol concentration (BAC). Additional MRS studies of alcohol in human and animal brains performed to date (Chiu et al. 1994; Mendelson et al. 1990; Moxon et al. 1991) support the finding that a lower alcohol signal exists in the brain than researchers would expect based on the BAC. Therefore, for some reason, the alcohol entering the brain is not fully visible using MRS.

The visibility of the signals in MR spectra is determined by the relaxation time of the nuclei being imaged. Consequently, the RF energy emitted by nuclei with short relaxation times may dissipate so rapidly that the signal is barely visible to the MR machine by the time RF detection begins. The signal is undetectable because it takes some time for the MR machine to apply a series of energy pulses and then to record the energy signal emitted by the nuclei in response.

**Figure 5:**
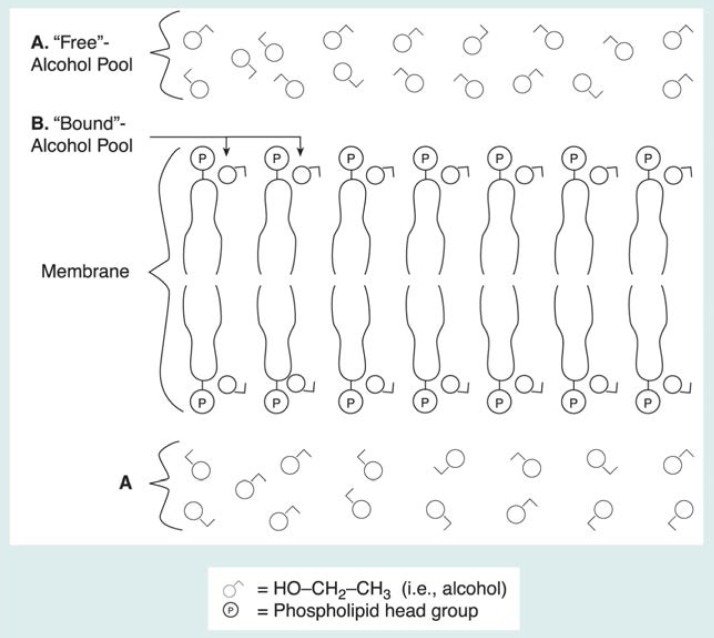
Researchers believe alcohol interacts with cell membranes in the brain, which contain relatively large molecules called phospholipids. These molecules are aligned so that the phosphorus-containing head groups (P) are at the surface of the membrane, which is arranged in a bilayer, and the long tails form the core of the membrane. (A) When alcohol enters the brain, it dissolves primarily in tissue water between and inside the cells (i.e., is a “free” pool of alcohol). (B) Some alcohol also binds to the membrane surface. Because the alcohol molecules interact with the membrane molecules, this pool of alcohol is restricted in its molecular mobility (i.e., is a “bound” pool of alcohol). The two alcohol pools create different types of signals in a hydrogen magnetic resonance spectrum, as described in the text and as shown in sidebar.

A short relaxation time may explain why only a portion of the alcohol signal is recorded. But why might only part of the signal be affected? Using the analogy mentioned earlier, a vibrating tuning fork’s energy dissipates quickly if the tuning fork is held rigidly and is not allowed to vibrate freely. It is the same for nuclei in tissues: They have long relaxation times if they are able to move around and rotate freely in their molecular environment (e.g., if they are in solution), but they have relatively short relaxation times if they are rigidly held in place (e.g., bound in cell membranes). Experiments with cell membranes isolated from their tissues suggest that brain alcohol exists in at least two pools: one containing alcohol molecules dissolved in water both outside and inside cells (i.e., a “free” pool of molecules not bound to any cell structures) (figure 5), which has a relatively long relaxation time, and one containing alcohol molecules bound to membranes (i.e., a “bound-alcohol” pool), which has a short relaxation time. The “free-alcohol” pool, which has the long relaxation time, is visible to MRS as a tall peak. In contrast, the “bound-alcohol” pool remains invisible, producing only a flat, broad signal indistinguishable from other signals in the MR spectrum. Furthermore, a rapid exchange of molecules may continuously occur between these pools. Evidence for the validity of this two-pool model is presented below.

## Differences in MR-Visibility in Alcohol-Tolerant vs. Nontolerant Subjects

**Figure 6:**
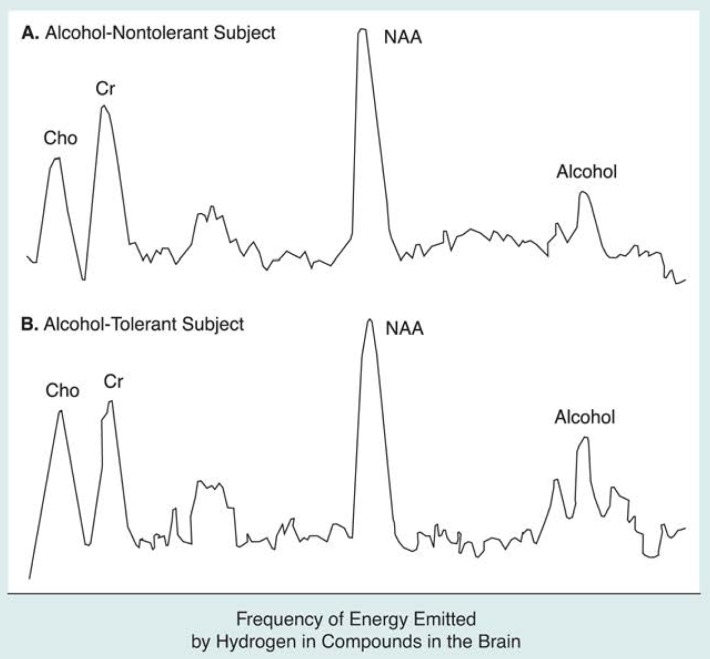
Hydrogen magnetic resonance spectra from (A) an alcohol-nontolerant and (B) an alcohol-tolerant subject demonstrate that less alcohol may be bound to brain cell membranes in people tolerant to alcohol’s effects. Signals (i.e., peaks) from the three major brain metabolites (i.e., Cho,^1^ Cr^2^ and NAA^3^) appear similar between the two spectra, but the alcohol signal in the nontolerant subject is smaller. Both spectra were obtained at similar BAC’s^4^ and at similar times after the subjects consumed alcohol. However, the brain alcohol concentrations represented by these spectra are 26 percent of BAC’s in the nontolerant subject and 52 percent of BAC’s in the tolerant subject. Because research indicates that both blood and brain alcohol concentrations are equivalent, the remaining alcohol in these subjects’ brains is “invisible” to MRS.^5^ The invisible portion, which is larger in the nontolerant subject, is the alcohol pool believed to be bound to brain cell membranes. ^1^Cho = choline-containing compounds; ^2^Cr = creatinine-containing compounds; ^3^NAA = N-acetyl-aspartate; ^4^BAC’s = blood alcohol concentrations; ^5^MRS = magnetic resonance spectroscopy. SOURCE: Adapted from Chiu et al. 1994.

An intriguing set of findings (Chiu et al. 1994) offers indirect support for the two-pool explanation of alcohol’s partial MR visibility. These findings report that brain alcohol is more MR visible (i.e., it forms a larger peak on the spectrum) in people who are tolerant to alcohol’s effects than it is in nontolerant people at the same BAC (figure 6). The study’s authors base their explanation on studies of cell membranes showing that the fluidizing effect of alcohol on membranes decreases when the membranes are treated with alcohol over a long period of time. In other words, exposing membranes to alcohol over a long period results in more rigid membranes than would exist if they were treated with alcohol over only a short period. The researchers hypothesize that, compared with nontolerant people, the greater membrane rigidity in alcohol-tolerant people allows less alcohol to bind to the membranes. A relatively greater alcohol concentration outside the membranes (i.e., MR-visible, or “free” alcohol) results, as does a relatively lower concentration of alcohol bound to the membranes (i.e., MR-invisible alcohol). If this model proves accurate, then the increased MR visibility of brain alcohol in alcohol-tolerant subjects may actually reflect an adaptation of the brain’s cells to alcohol.

Other researchers have found supporting evidence that MRS visibility of alcohol increases under conditions that result in short-term (i.e., acute) alcohol tolerance.^4^ For example, Mendelson and colleagues (1990) found that an increase in MRS brain alcohol visibility occurred between 1 and 2 hours after a single dose of alcohol was consumed. Moxon and colleagues (1991) observed dramatically increased MRS brain alcohol visibility in dogs that were given additional doses of alcohol within 30 minutes of the initial dose. Most recently, Kaufman and colleagues (1996) demonstrated increased MRS visibility of brain alcohol with repeated alcohol administration in humans during a single test session, results which may reflect acute tolerance.

## A Possibly Related Phenomenon Observed Using Phosphorus MRS

As previously mentioned, MRS also can create spectra from energy emitted by compounds containing atoms other than hydrogen. For example, phosphorus combines with fats, or lipids, to form phospholipids, a primary building block of cell membranes. Thus, phosphorus MRS can be used to evaluate changes in cell membranes among subjects who have a disease or a condition such as tolerance resulting from chronic alcohol abuse.

Phosphorus MRS of living subjects also allows for the observation of compounds in cells called phosphodiesters (PDE’s). The PDE signal in a phosphorus spectrum originates from three components, each of which can provide information about changes in cell structure or activity. There is (1) a long relaxation-time component derived from the products of the breakdown (i.e., metabolism) of phospholipids; (2) an intermediate relaxation-time component derived from phospholipids in compartments inside cells (i.e., vesicles); and (3) a short relaxation-time component derived from intact phospholipids in membranes (Kilby et al. 1991). The relaxation times are shorter for the second and third components because they are bound in cell structures.

Meyerhoff and colleagues have used phosphorus MRS to study the effects of chronic alcohol abuse on phospholipids (Meyerhoff et al. 1995*a*). Experimental requirements for phosphorus MRS (i.e., a delay of 2 milliseconds [ms] or less between RF excitation and detection by the MR instrument) make observation of at least part, if not all, of the intermediate and short relaxation-time pools feasible (Murphy et al. 1989). In contrast, hydrogen MRS, which requires a delay on the order of at least 20 ms between excitation and detection of RF energy, cannot directly detect the short relaxation-time pool of brain alcohol, as described earlier.

The primary finding from Meyerhoff and colleagues’ (1995a) research supports Chiu and colleagues’ evidence (presented earlier) of cell membrane changes resulting from long-term alcohol abuse. Meyerhoff and colleagues showed that the intensity of the PDE signal was reduced in chronic alcohol abusers. Similar to the findings of increased alcohol MR visibility among alcohol-tolerant people, this reduction in signal intensity most likely results either from a lowered concentration of PDE’s or from a shortened average PDE relaxation time, which causes a smaller PDE signal magnitude. Recent biochemical studies suggest that PDE concentrations are not reduced among chronic alcohol abusers, so a shortened relaxation time for the reduced PDE signal intensity appears to be the most favorable explanation.

Meyerhoff and colleagues thus hypothesize that the observed reduction of the PDE signal in alcohol abusers reflects a reduction in relaxation time for the intermediate and short T_2_ PDE pools, as a result of increased membrane rigidity. This model involves the same mechanisms thought to underlie the increased brain alcohol visibility to hydrogen MRS in alcohol-tolerant people (Chiu et al. 1994). In other words, the changes in phosphorus MRS observed in chronic alcohol abusers may reflect the same underlying membrane changes (i.e., increased membrane rigidity resulting from alcohol tolerance) that are hypothesized to cause increased hydrogen MRS visibility of brain alcohol. Phosphorus MRS also demonstrates increased cell membrane rigidity without requiring the subject to consume alcohol before imaging, suggesting that the rigidity is at least a semipermanent condition in the chronic alcohol abuser.

## Testing the Model of Alcohol Magnetic Resonance Visibility

Although there is a possibility that an “MR-invisible” pool of alcohol exists in the brain after consumption, this hypothesis is more complex than the description presented earlier implies. Alcohol in a short relaxation-time pool, such as the hypothesized pool bound to cell membranes described earlier, would produce a flat, broad component in the MR spectrum if it existed (see sidebar, p. 313). In essence, this flat component is invisible in MRS studies because it cannot be differentiated from signals produced by other compounds or from noise (i.e., signals) inherently created by the MR procedure. Nevertheless, researchers can obtain indirect evidence for the existence of this broad, flat signal using a method called magnetization transfer (MT). MT selectively cancels the shallow signal produced by alcohol in the pool bound to cell membranes. Because alcohol molecules are constantly shifting between the free and bound pools, this cancellation is transferred, in part, to the free-alcohol pool, visibly diminishing the free-pool signal in an MR spectrum.

**Magnetization Transfer and Off-Resonance Saturation**

**Figure:**
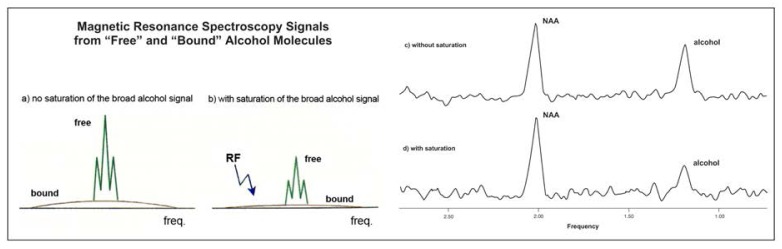
Schematic drawing showing two magnetic resonance spectra from alcohol in the brain (a) and the effect of an off-resonance energy saturation pulse on the two signals produced by alcohol (b). Experimental data (c and d) were obtained from a rat after it had been administered alcohol. (c) The spectrum shows a normal peak produced by the brain chemical NAA^1^ and a peak from alcohol. (d) An off-resonance saturation pulse was applied, diminishing the peak produced by alcohol but not that produced by NAA. This is indirect evidence of a pool of alcohol invisible to magnetic resonance, possibly bound to brain cell membranes. ^1^NAA = N-acetylaspartate.

Magnetization transfer (MT) is a phenomenon that allows researchers to obtain indirect evidence for portions, or pools, of a compound (e.g., alcohol) that otherwise would not be observable using magnetic resonance spectroscopy (MRS). Researchers have hypothesized that alcohol exists in the brain in two pools: a “free” pool, in which alcohol molecules are not bound to any tissues, and a “bound” pool, in which alcohol molecules are bound to cell membranes. To obtain evidence that some alcohol may be invisible to MRS, investigators must somehow alter the MRS signals from visible pools. As shown in part (a) of the figure, the bound pool emits a broad, shallow signal that is essentially invisible on MRS. This broad signal is thought to have the same central frequency as the signal produced by the free-alcohol pool, which appears as a tall triplet peak at the center of the bound pool’s broad signal. Researchers can use the interactions that occur between the two alcohol pools to establish the existence of the invisible pool. One such interaction is a rapid exchange of alcohol molecules between the pools.

An MRS experiment has been designed that cancels the broad signal of the bound pool and indirectly affects the signal from the free pool. The experiment uses a technique called off-resonance saturation, in which the magnetic resonance machine applies energy to the tissue at a certain frequency different from that of the free pool (i.e., an off-resonance saturation pulse). The off-resonance pulse eliminates the bound pool’s broad, shallow signal but does not cancel the free signal (part [b] of the figure). This task is simple because of the broad frequency range produced by the bound-alcohol pool. The effect produced reduces the size of the free-alcohol peak, because bound molecules affected by the off-resonance energy move from the bound to the free pool during the natural process of molecular exchange, reducing the size of the free signal.

The production of this off-resonance saturation, or MT, effect for a specific compound provides indirect evidence of the existence of a broad signal for that compound (parts [c] and [d] of the figure). In other words, if the bound-alcohol pool does not exist or if a rapid exchange of alcohol does not occur between the free and the bound pools, the off-resonance saturation pulse would not affect the size of the alcohol peak in the spectrum. Conversely, if the bound-alcohol pool does exist and rapid exchange does occur, the visible alcohol peak will decrease in size. Differences in the size of the off-resonance saturation effect, as evidenced by differences in the height and width of the visible alcohol peak between conditions (e.g., alcohol-tolerant versus nontolerant subjects), can be viewed as indicators of differences in the relative size of the bound-alcohol (i.e., the magnetic resonance-invisible) pool.

— George Fein, Dieter J. Meyerhoff, and Michael W. Weiner

In recent MT experiments in rats that had been administered alcohol, Meyerhoff and colleagues (1995b) found a significant reduction (about 40 percent) of the visible alcohol signal after they applied the MT technique (see figure in sidebar). Because it is consistent with the findings presented earlier, this MT effect suggest that a short relaxation-time alcohol pool exists and supports the theory that this pool transfers energy during MT to the long relaxation-time pool. Similar experiments are planned in humans to test the hypothesis that bound- and free-alcohol pools exist in the human brain. Moreover, such experiments can be used to test the model put forth by Chiu and colleagues regarding their findings of increased alcohol visibility in alcohol-tolerant individuals. If the bound-alcohol pool in alcohol-tolerant subjects is smaller than that of alcohol-nontolerant subjects, the MT effect should be smaller in alcohol-tolerant subjects.

## Implications and Future Directions

The phenomena and methods described in this article may be used in studies of alcohol’s effects on the brain as well as of individual differences in the response to alcohol and the vulnerability to alcohol abuse. For example, researchers imaging alcohol in the brain can determine whether alcohol shows an affinity for specific brain regions or is distributed uniformly within a tissue type. The concentration of alcohol in certain brain areas may help to explain some of its effects on people’s behavior. Imaging of brain alcohol also could help determine whether the brain distribution of alcohol differs by gender or changes with age or in response to particular diseases. Such studies could help elucidate any physiological mechanisms that may underlie such differences.

Regarding alcohol’s partial MR visibility in the brain, basic studies are needed to make the methods as reliable as possible and to replicate the results described earlier. With these goals accomplished, the potential yield from brain alcohol MRS and MRSI studies will be enormous. Foremost, the differences seen between the MR spectra of people who are tolerant and those who are not tolerant to alcohol’s effects may be a powerful in vivo measure for assessing cellular tolerance to alcohol in the brain. This measure could then be used to determine how long such tolerance persists after a person becomes abstinent and whether the persistence depends on such parameters as the amount and timespan of alcohol use, the subject’s age, and genetic factors. The greater visibility of brain alcohol in alcohol-tolerant people may even reflect a genetic vulnerability to alcohol abuse. In other words, genetic predisposition to alcohol abuse may manifest itself in increased rigidity of cell membranes in the brain even among people who have never consumed alcohol. This theory can be tested using MRS methods by comparing the brain-alcohol visibility of people with a family history of alcohol problems with that of people with no family history of alcoholism. These methods also could be employed to study subjects’ responses to acute doses of alcohol and stimuli that precipitate a craving for alcohol. Finally, if the phenomenon of increased visibility of brain alcohol in alcohol-tolerant people is verified, other MR-observable physiological signs—like those described with phosphorus MRS—must be sought that may reflect the phenomenon without subjects’ having to consume alcohol. For example, if the partitioning of brain water into bound and free pools is similar to that of brain alcohol, it may be possible to devise water MRI–MT studies that can assess the same phenomena that are now studied using hydrogen MRS after alcohol administration. Although such a day is a long way off, if such measures become feasible, they may be of clinical value in monitoring recovery from alcohol abuse.

## Footnotes

1 A nerve cell comprises a body—which contains the cell nucleus and other essential structures—and incoming and outgoing projections, or fibers. Gray matter in the brain is made up mostly of cell bodies and many tiny branches of incoming fibers (i.e., dendrites) that bring signals from other nerves to the cell body. Each nerve cell generally also has one large outgoing fiber (i.e., the axon) that conducts nerve signals to other nerves. White matter is made up mostly of these axons.

2 The areas outlined by the peaks also depend on the volume of tissue studied, the rate at which RF energy dissipates in the compounds, and the interval between when the tissue absorbs energy and when the measurement for the spectrum is taken.

3 MR experiments involve other relaxation-time phenomena (called T_1_ and T_2_^*^ [i.e., T_2_ “star”] relaxation times), but T_2_ relaxation is the critical phenomenon involved in the capacity of MRS to detect alcohol in the brain.

4 Acute tolerance to the effects of alcohol occurs during a single drinking episode and can be demonstrated by measuring a person’s physical coordination in relation to BAC. Over time, the BAC rises, peaks, and falls again. A person’s lack of coordination is greater when the BAC is rising (i.e., early in the drinking episode) than when the BAC is falling (i.e., later in the drinking episode). This is true even when coordination is measured at the same BAC during both the rising and falling phases. The person has developed a short-term, or acute, tolerance to the physiological effects of alcohol.
